# Spawning Coordination of Mates in a Shell Brooding Cichlid

**DOI:** 10.1155/2012/517849

**Published:** 2012-08-09

**Authors:** Dolores Schütz, Zina Heg-Bachar, Michael Taborsky, Dik Heg

**Affiliations:** ^1^Department of Behavioural Ecology, Institute of Ecology and Evolution, University of Bern, Wohlenstr 50a, 3032 Hinterkappelen, Switzerland; ^2^Division of International and Environmental Health, Institute of Social and Preventive Medicine (ISPM), University of Bern, Finkenhubelweg 11, 3012 Bern, Switzerland; ^3^Clinical Trials Unit Bern, Department of Clinical Research, University of Bern, Finkenhubelweg 11, 3012 Bern, Switzerland

## Abstract

*Aim*. External fertilisation requires synchronisation of gamete release between the two sexes. Adequate synchronisation is essential in aquatic media because sperm is very short-lived in water. In the cichlid *Lamprologus callipterus*, fertilisation of the eggs takes place inside an empty snail shell, where females stay inside the shell and males have to ejaculate into the shell opening. This spawning pattern makes the coordination of gamete release difficult. *Methods*. This study examined the synchronisation of males and females during egg laying. *Results*. The results showed that the male initiates each spawning sequence and that sperm release and egg laying are very well synchronised. 68% of all sperm releases occurred at exactly the same time when the female laid an egg, and 99% of ejaculations occurred within ±5 seconds from egg deposition. On average 95 eggs are laid one by one with intervals of several minutes between subsequent eggs, leading to a total spawning duration in excess of six hours. *Conclusions*. We discuss this exceptional spawning pattern and how it might reflect a conflict between the sexes, with males attempting to induce egg laying and females extending the egg laying period to raise the chance for parasitic males to participate in spawning.

## 1. Introduction

In species with external fertilisation, males and females must synchronize gamete release. In fish, various forms of information that transfer between the sexes are involved to ensure fertilisation. Visual communication involves the use of colour signals [1–3] and behaviour [4, 5]. Chemical signals may be used to coordinate spawning, as fish possess a very powerful olfactory apparatus detecting odours in very low concentrations [6–8]. Some fish use also sound production during courtship and spawning [9–11].

In the highly polygynous cichlid, *Lamprologus callipterus *(Boulenger), fertilisation of eggs takes place inside an empty shell of the snail *Neothauma tanganyicense*. Large nest males collect these shells as spawning substrate and defend shell nests as their territories [12]. Females ready to spawn visit these nests, select a shell, spawn inside of them, and take care for the brood for 10 to 14 days [13]. Among all animals, this fish species shows the most extreme sexual size dimorphism (SSD) with males being larger than females. In our study population, nest males are on average 10 times heavier than the females they spawn with, but the magnitude of SSD varies greatly between populations, with up to 60 times heavier males than females in Muzimo, a Northern population in the Democratic Republic of Congo [14]. Results of an earlier study suggest that sexual selection mechanisms are probably not as important as natural selection mechanisms for the evolution and maintenance of the SSD in *L. callipterus* [15]. Rather, this extreme SSD appears to be mainly affected by ecological constraints, with opposing selection pressures on the two sexes: males need to pass a threshold size to be able to carry shells, and female size is constrained by the limited size of their breeding substrate, shell size, and by intrasexual competition for shells ([14, 15], see also [16]).

During spawning, males and females have highly restricted visual contact and hardly any direct bodily contact, because the female head sticks deep inside the shell and the male is much too large to enter the shell [13, 15]. On average 91 eggs are laid (range: 35–160, data from ref. [17]), and laying of a whole clutch lasts exceptionally long. In an earlier laboratory experiment, spawning took 9.3 h (range: 5.5–12 h) and in the field 6.9 h (*n* = 29 spawnings at 10 nests, range: 2.16–10.28 h, [18]). Nest male spawning may be parasitized by two other male types performing alternative mating tactics, medium sized sneaker males and dwarf males, which can reside inside the shell during the course of spawning [12, 19, 20]. Parasitic males are always exposed to sperm competition, whereas nest males monopolize spawning without the participation of reproductive parasites in most spawnings [12, 21]. Preliminary results suggest that nest males' sperm lived longer than dwarf male sperm due to the longer sperm head size, but that dwarf male sperm swam straighter and faster than nest male sperm [22]. Little is known about the different spawning behaviours of the two sexes in this species. 

Previous work on male-female timing and coordination of spawning behaviour has focused on species in which males and females have full visual and often also bodily contact [23, 24]. This is the first study addressing such male-female interactions where visual and bodily contact is highly limited. Using field and laboratory experiments we examined who is initiating a spawning bout and whether and how mating pairs of *L. callipterus* mutually synchronize gamete release under the limited availability of visual information. 

## 2. Methods

### 2.1. Behaviours

In the field, the following behaviours of males and females were recorded without the observers entering their nests or disturbing the spawning process: male mouthing, female shifting, and male moving forward to put his genital papilla motionless over the shell entrance, which equals the duration of sperm release [18] (Table 1). In the laboratory, the behaviour and location of males and females were recorded simultaneously, including male head shake, male head in, female egg laying, and female moving out of the shell. Distinct behaviour events were separated from each other either by inactivity or other behaviours. Behaviours in the laboratory were recorded as frequencies (event) or durations (state) with “the Observer 3.0” (Noldus Information Technology).

### 2.2. Field Observations

Field data were collected in May 2002 at Kasakalawe, Lake Tanganyika, Zambia (8°46′S; 31°5′E) by Scuba diving at a depth of 12.8–15.9 m. The colony consisted of more than 100 nests, 97 of which were marked with numbered stones and observed in total 250 times. Spawning males were detected by hovering about one meter over the colony and observing each nest for 5 min. If spawning was detected, this nest was observed for 10 to 20 minutes (mean observation duration: 11.52 minutes), and the behaviours of all participants were recorded. Analyses were done at the female level to avoid pseudoreplication.

#### 2.2.1. Mouthing Experiment

The male's opening and closing of the mouth when his head sticks into the shell entrance produce a water flow that can be detected by the female [25]. Therefore, we hypothesized that by mouthing a male might stimulate the female to lay an egg, and if she reacts, release sperm. To test this hypothesis, the mouthing was imitated experimentally. Twenty spawning and 165 guarding females were tested to check whether they reacted differently to this potential signal. Females of *L. callipterus* never hide in snail shells from predators [13] and no female, that is not ready to spawn, already spawning, or guarding, is accepted in the territory by the nest owner. Therefore, females staying inside snail shells are all guarding females and not individuals that simply hide in shells. We predicted that spawning females should react with shifting to prepare egg release, while guarding females should not show such behaviour. As spawning females, we used females that were currently laying eggs in shells of nest males, and spawning always restarted within 3 minutes after our short disturbance. As guarding females, we used females that currently took care for their broods in a nest male's shell, also without influencing their natural behaviour any further. Each of the groups was treated in two different ways in randomised succession: in the experimental treatment, male mouthing was simulated with our fingertips by creating a water current into the shell entrance. In the control treatment the fingertips were held still over the shell entrance. For each group of females, half of the fish (determined randomly) started with the experimental treatment and the other half with the control treatment, and female reaction to the manipulation was recorded. Shifting behaviour looks exactly the same in the situation when the water current is produced by a mouthing male versus when the water current is produced by moving fingers. We could not standardize the water current between traits completely. However, one person stimulated the mouthing behaviour in all trials (ZHB) and attempted to keep the water current as constant as possible between trials. A hierarchical log-linear analysis with backward elimination of terms was performed to test whether the group (guarding or spawning females), the treatment (control or experimental), or their interaction influenced the female's reaction (yes: female shifting; no: no reaction). 

#### 2.2.2. Undisturbed Observations

During 65 observed spawning events within 40 nests, the frequency of the nest male mouthing the shell with a spawning female inside was determined. Subsequently, it was noted whether the female showed shifting behaviour and whether the male released sperm. Mouthing was also observed at shells with non-spawning (guarding) females inside (*n* = 140), and the reaction to male mouthing behaviour was compared between spawning and guarding females. The occurrence of different sequences of different spawning behaviours was analysed with *χ* ^2^-tests.

### 2.3. Laboratory Experiment

For the laboratory experiment, shells were prepared with Plexiglas windows to allow the observation of egg laying without disturbing the animals. Spawning observations were performed in four 100-litre tanks, into each of which five empty *N. tanganyicense* shells were introduced that were fixed to a PVC plate with silicon glue. Three of the five shells were closed with a small stone to prevent females from entering. The two other shells were positioned with their Plexiglas windows against the front screen of the tanks to enable video recording. Into each tank, a nest male (SL range: 89–123 mm) and five adult females (SL range: 41–50 mm) were introduced. In all four tanks, the two visible shells were continuously recorded on videotape for 13 hours a day (from 08:00 hours to 21:00 hours). These recordings were analysed for all periods during which a female was inside a shell and then started spawning. 

#### 2.3.1. Analysis of Laboratory Data

Nine spawning events could be used to analyse all behaviours involved in egg laying and sperm release. Since males released sperm already before the female started to lay eggs, the time the male released sperm for the first time and the time until the female laid her first egg (“prelaying period”) were determined. The time for which males continued to release sperm after the female stopped laying was also determined (“postlaying period”). Male sperm release frequencies and durations between the pre-, post-, and egg-laying periods were compared. Descriptive statistics show means ± SD if data were normally distributed (Kolmogorov Smirnov-tests, *P* > 0.1) and medians with quartiles if data differed from a normal distribution (*P* < 0.1). Note that period durations and behaviour traits varied widely between females, and some periods were missing for some females due to impeded sight. Therefore, we appropriately used paired *t*-tests (per female) to test for male differences in sperm release characteristics between the pre- and laying periods, rather than ANOVA to test the differences among three periods at once. For the pre-and postlaying periods, *n* = 6 spawnings, because three females were already inside the shell when recording started (but had not started egg laying yet), and for three females, the recording ended before the male had ceased sperm release in the postlaying period. For the laying period, all 9 spawnings could be used for data analysis of sperm release frequency and duration, but only 6 cases for comparing period durations (pre- versus egg-laying versus postperiod).

To test whether and how male and female spawning patterns were synchronised, we analysed for each egg laid the behaviours shown in the period ranging from 15 sec before until 15 sec after it was laid (31 seconds, where 0 = −0.5 to +0.5 sec around the egg being laid). Fourteen cases were observed where two eggs were laid and one case where three eggs were laid in one spawning bout, that is, within 15 seconds after the first egg was laid. Here, behaviours were analysed around the timing of the first of these multiple eggs. The proportion of time males and females showed different spawning behaviours within these 31 seconds around egg laying and the median time differences of each behaviour to the moment of egg laying were determined. The relationship between the number of eggs laid and the number of ejaculations was determined, as were the time intervals between two laid eggs and two subsequent sperm releases. Additionally, it was checked whether and how the intervals between two subsequent eggs, between two sperm releases, and the sperm release duration varied between pairs and within the egg laying period. All analyses were performed by SPSS and report two-tailed probabilities. *χ* ^2^-values are from chi-square cross-tabulation tests, unless otherwise specified.

## 3. Results

### 3.1. Field Observations, Mouthing Experiment

**Figure 1:**
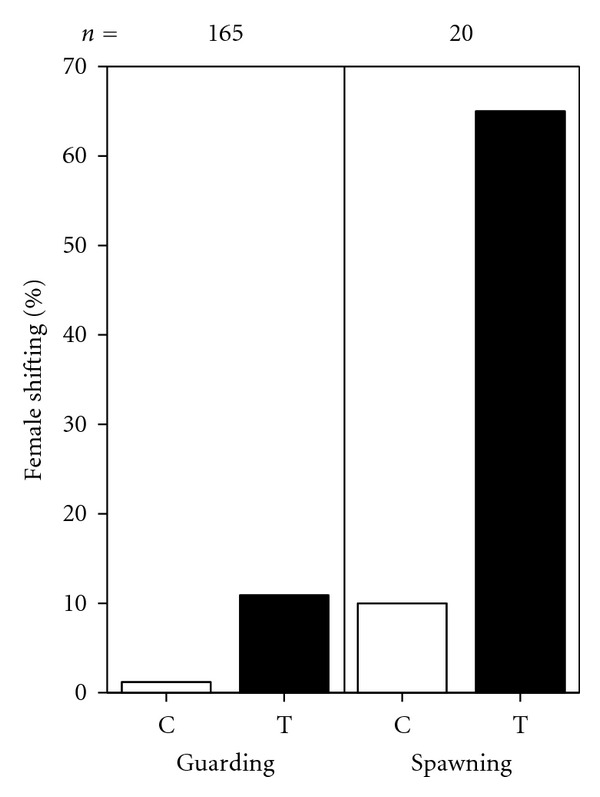
Percentage of cases in which guarding and spawning females reacted by shifting to the control, no finger movements (C), and experimental treatments, water current induced by finger movements (T).

As expected, in the field spawning females reacted significantly more often to the experimental treatment (simulating mouthing behaviour) by shifting behaviour than guarding females (hierarchical loglinear analysis with backward elimination of nonsignificant terms: group × reaction: *χ* ^2^ = 27.8, *P* < 0.0001, Figure 1). Additionally, experimentally tested females shifted significantly more often than females of the control treatment (treatment × reaction: *χ* ^2^ = 25.8, *P* < 0.0001, Figure 1). Other interactions were all nonsignificant (group × treatment: *χ* ^2^ = 3.8, *P* = 0.059, group × treatment × reaction: *χ* ^2^ = 0.198, *P* = 0.656). Note that moving our fingers to the shell entrance (control) and subsequently induction of the water current (treatment) never induced the females in any of the 2 × 2 treatment arms to retreat into the shell to flee from this disturbance.

### 3.2. Field-Undisturbed Observations

In the field, out of seven possible behaviour sequences of male mouthing, female shifting, and sperm release, only three were actually shown (see Table 2 for frequencies of sequences). It never occurred that the female shifted and/or the male released sperm without male mouthing behaviour shown before. In 110 of the 796 observed mouthings, a rapid sequence of mouthing with no reaction was shown, followed quickly again by mouthing, female shifting, and sperm release. Altogether, female shifting occurred 473 times and sperm release occurred 476 times after 796 mouthing events (three times the male released sperm after mouthing without the female showing shifting behaviour in between). Therefore, female shifting and male sperm release showed almost the same frequency and always followed mouthing, but 40% of mouthings did not result in spawning. Mouthing was also performed at shells with guarding females inside (*n* = 140), but guarding females never responded by any behaviour. The reactions to male mouthing behaviour differed highly significantly between spawning and guarding females (*χ* ^2^ = 168.2, df = 1, *P* < 0.001).

### 3.3. Laboratory Experiment

#### 3.3.1. Timing of Female Egg Laying and Male Ejaculation

**Figure 2:**
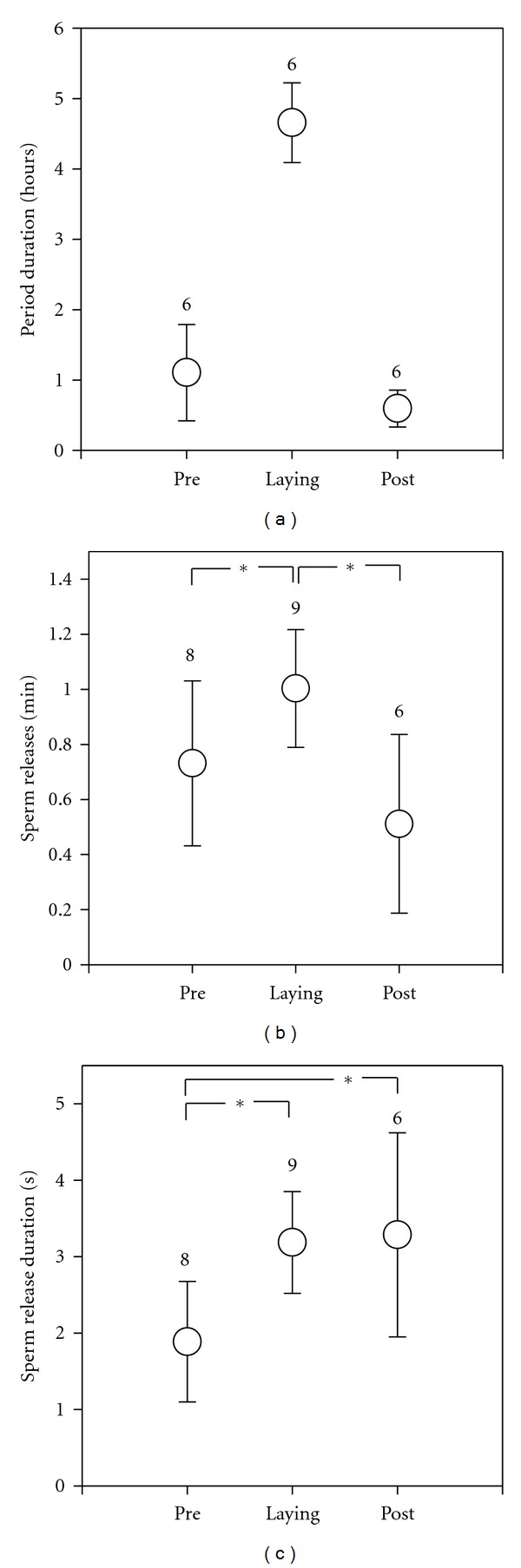
(a) Durations of the prelaying, laying, and postlaying periods in hours; (b) numbers of sperm releases per minute; (c) sperm release durations in seconds. Depicted are means and standard errors of the mean, numbers indicate sample sizes, *mark significant differences *P* < 0.05.

During nine observed spawning events of an entire clutch, females laid the first egg 66.3 ± 41.0 min (mean ± SD, range: 31.7–110.1 min) after entering the shell for spawning (prelaying period, see Figure 2(a)). Egg laying lasted 279.6 ± 34.2 min (*n* = 9, range: 242.4–326.4 min, laying period), during which an egg was laid on average every 2.14 min (0.47 ± 0.37 eggs laid per min). Males started to release sperm long before the female laid her first egg, about four minutes after the female went into the shell (median = 3.98, quartiles: 1.29–9.60, range: 0.08–68.53 min). Males continued to ejaculate after the female laid her last egg on average for 35.7 ± 15.8 min (*n* = 6, range: 16.1–57.9 min; i.e., post laying period, Figure 2(a)). Ejaculation rates were significantly lower during the pre- and postlaying periods compared to the laying period (paired *t*-tests; prelaying versus laying period *t*_7_ = −2.8, *P* = 0.026; laying versus postlaying period *t*_5_ = −4.4, *P* = 0.007, Figure 2(b)). Sperm release lasted significantly shorter during the prelaying period than during the laying and postlaying periods (paired *t*-tests; prelaying versus laying period *t*_7_ = −4.5, *P* = 0.003; prelaying versus postlaying period *t*_4_ = −4.6, *P* = 0.01, Figure 2(c)), whereas there was no difference between the postlaying versus the laying period.

#### 3.3.2. Synchronisation between Males and Females

**Figure 3:**
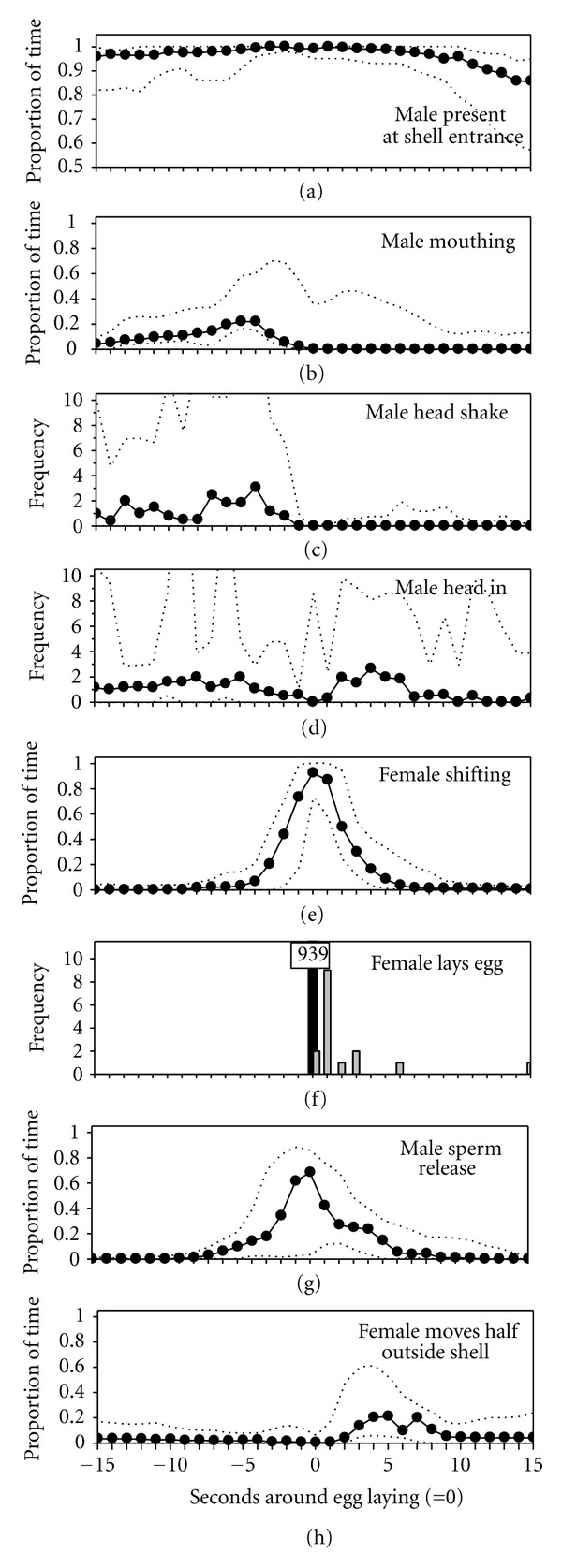
Proportions of time and behavioural frequencies during 31 one sec intervals around 939 eggs laid of (a) the male being present at the shell, (b) the male showing mouthing behaviour, (c) male “head shake” behaviour, (d) male “head-in” behaviour, (e) female shifting, (f) egg laying (*n* = 939: black bar); note that in 14 of 939 cases a second (*n* = 14 eggs) or a third egg (*n* = 2 eggs) was laid within 15 sec (grey bars, total *n* = 955 eggs). (g) Sperm release, (h) female moving half outside the shell. All panels show medians (black dots) and the total range (dotted lines) for *n* = 939 eggs.

Synchronisation between males and females was very high: males spent significantly different proportions of time at the shell within the 15 sec before and 15 sec after the deposition of an egg (Friedman test, *χ* ^2^ = 194.4, df = 30, *P* < 0.001, Figure 3(a)), but when the female laid an egg, the nest male was almost always present at the shell. Out of 902 eggs laid, only 10 were laid with the males ejaculating more than 5 sec before or after the eggs were laid. Also the frequencies of male mouthing behaviour, male head shaking, and male head in varied systematically around egg laying (Friedman tests, 77.3 < *χ* ^2^ < 189.4, df = 30 in each test, *P* < 0.001 for each behaviour, Figures 3(b)–3(d)). Briefly before egg deposition females showed shifting behaviour (median = −1.6 sec, Friedman test, *χ* ^2^ = 202.9, df = 30, *P* < 0.001, Figure 3(e)). 68.3% of all sperm releases occurred at exactly the same time when the female laid an egg, and in 98.9% of all cases, the male released sperm between 5 sec before and 5 sec after egg laying (Friedman test, *χ* ^2^ = 211.9, df = 30, *P* < 0.001, Figure 3(g)). On average 2.26 (±0.7 SD) ejaculations occurred per laid egg. After egg deposition, the female often moved briefly partly out of the shell (Friedman test, *χ* ^2^ = 167.7, df = 30, *P* < 0.001, Figure 3(h)). The interval between two subsequent egg depositions (mean ± SD: 128 ± 101.5 sec) was on average more than twice as long as the interval between two sperm releases (mean ± SD: 62.5 ± 58.2, Wilcoxon's paired test, *z* = −2.666, *P* = 0.008), but both intervals were highly variable. 

#### 3.3.3. Behavioural Changes during Laying of a Clutch

**Figure 4:**
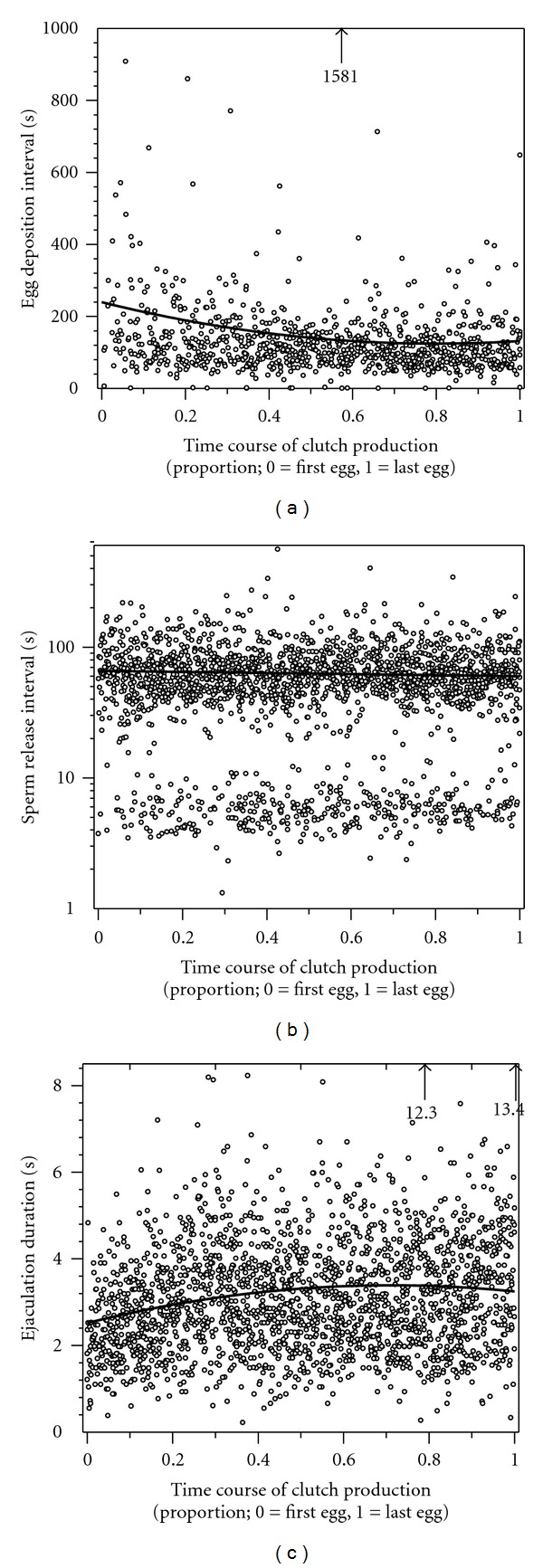
(a) Egg deposition interval, (b) sperm release interval (on a log scale), and (c) sperm release duration in the time course of producing a clutch (0 = first egg laid, 1 = last egg laid). Black lines show the fitted mean values for the 9 spawnings observed (see Table 3), and numbers point towards outliers.

With increasing laying duration, females laid the eggs more quickly, but towards the end of laying the egg deposition rate slowed down (Table 3: egg interval, Figure 4(a)). Double eggs and triple eggs (see Figure 3 for definition) occurred evenly distributed over the spawning period (logit General Linear Model, effect of the time course of clutch production *P* = 0.64). The intervals between subsequent sperm releases also varied systematically over the laying period of a clutch (Table 3: ejaculation interval, Figure 4(b)). In seven pairs the sperm release intervals decreased during laying, but in two pairs they increased. The sperm release duration differed significantly between males and generally increased during the laying of a clutch, before slightly decreasing again at the end of laying (Table 3: sperm release duration, Figure 4(c)).

## 4. Discussion

Despite the limited communication possibilities during spawning, the synchronisation between sperm and egg release is very high. The male signals his readiness to spawn to the female by mouthing into the shell entrance. If the female is ready to spawn she responds to male mouthing by shifting (as confirmed in the mouthing experiment), after which she deposits an egg (median = 1.6 sec after shifting). Egg laying appears highly synchronised with male sperm release (median 0.2 sec after female shifting): in 98.9% of all cases, male sperm release occurred between 5 sec before and 5 sec after egg laying. Good synchronisation is important mainly for three reasons. First, sperm are very short lived in fresh water, so they should meet with the egg within about a minute [22]. Second, female *L. callipterus* usually lays one egg at a time, with long intervals between successive eggs, so each egg needs to be fertilized separately. Third, the prevalence of sperm competition in this species, where dwarf males can reside inside the shell during the course of spawning, which should raise the importance of spawning coordination with their female, at least from the perspective of nesting males [12, 19, 20].

These results show that males and females intensely communicate to synchronise their gamete release inside the snail shell. Apparently, this involves mainly behavioural cues like in the lekking cichlid *Lethrinops parvidens*  (Trewavas; [26]) and the Atlantic cod *Gadus morhua* L. [27]. The field experiments suggested that “mouthing” is a crucial male behaviour to induce female egg laying. In the mouthing experiment, spawning females reacted significantly more often to the experimental treatment (simulating mouthing behaviour) by shifting behaviour than guarding females. One might argue that guarding females may detect the water current as signal of predators that try to enter shells. Then, they should not react to the created water current, which also possibly results in the different reactions between spawning and guarding females. However, predators are hardly ever able to perform behaviours similar to male mouthing behaviour above a shell in the field, because the nest male rigorously defends his territory against intruders. Moreover, guarding and spawning females react to a major disturbance (e.g., us touching the shell) by moving and hiding deep inside the shell and then freeze. This is completely different from shifting behaviour. We never observed females to retract into the shell due to the presence of our finger tips at the shell entrance in either treatment. Thus, we do not think that guarding females differently perceived the imitated mouthing behaviour and therefore reacted differently than spawning females.

 In undisturbed observations in the field, out of seven possible sequences of important male and female behaviours at spawning, only three occurred, with the sequence “male mouthing—female shifting—sperm release” being most frequent (see Table 2). Female shifting and male sperm release occurred at almost the same frequency (about every 2 min), while mouthing was displayed almost twice as often. Apparently, males attempt to stimulate females by mouthing, and if they react, males respond by releasing sperm. 

Males began to ejaculate already in the first five minutes after the female entered the shell for spawning, but females waited more than an hour before laying their first egg. This may suggest that male ejaculations are required for an extended period of time before females start laying, so this may be regarded as part of their courtship. Males might need to signal the availability, quantity, or quality of ejaculates to the female to induce egg deposition. However, given that males suffer from sperm shortage at late stage of spawning [18], prolonged ejaculation in the prelaying period will reduce fertilization success. Then, females may not necessarily prefer ejaculation in the prelaying periods, particularly if such ejaculates result in reduced fertilization success due to sperm shortage, and males may not release sperm in this stage. Males also continued to ejaculate for more than half an hour after the female had laid her last egg. This could be due either to limited information of the male that the female has stopped laying eggs, or it could reflect attempts of the male to induce the female to lay more eggs (see below). 

During the egg laying phase in the laboratory experiment, males ejaculated on average more than twice as often as females deposited an egg. Both in the field and in the laboratory, females deposited one egg about every other minute. In the field, the interval between two ejaculations was almost the same as the egg-laying interval, but in the laboratory it was only about half as long. This discrepancy might be due to the higher sperm availability of males in the laboratory, allowing them to challenge their partner to spawn more often. In the field, males spawn with up to 4 females simultaneously (unpublished data), and sperm shortage seems to be a limitation for nest male reproduction [18]. Male timing of sperm release showed two interesting patterns (see Figure 4(b)). First, males often perform a second sperm release shortly after the first one (3–10 sec later, exemplified by the lower band of cases in Figure 4(b) around 3–10 sec). Second, males almost invariably spawned simultaneously with egg release and once again halfway between two eggs (40–140 sec later, exemplified by upper band of cases in Figure 4(b) around 40–140 sec). The second sperm releases were often performed without the female reacting to the males' mouthing behaviour.

The total spawning duration of a clutch was 6.3 hours in the lab (from the first until the last sperm release), during which time the females laid one egg approximately every 2 minutes within 4.55 hours (0.47 eggs/min). In other cichlid species, spawning lasts much shorter, for instance about 1 hour in the mouth-brooder *Tramitichromis intermedius*  (Trewavas; [3]), in *Neolamprologus pulcher*  (Trewavas & Poll; own observations), and in *Neolamprologus leleupi*  (Poll; [28]), during which time the latter two species may lay more than 150 eggs. Longer spawning durations of 2 to 3 hours have been recorded in *Neolamprologus hecqui*  (Boulenger; [28]) and *Cichla monoculus*  (Spix & Agassiz; [29]), during which time these species lay usually less than 100 eggs. Therefore, it seems that the long spawning duration per clutch of more than 6 hours in *L. callipterus* is exceptional among cichlids and perhaps in fish in general. A possible function of this extended spawning period might be the induction of sperm competition by females (cf. [30]). There is increasing evidence that better sperm competitors sire higher quality offspring [31–33]. By extending the spawning duration females can increase the chances that other males participate in fertilising the eggs, which seems particularly appropriate in a species with two different male parasitic spawning tactics. Females may even prefer to spawn with many males, independently of sperm competition, because sperm shortage may cause reduced fertilization success of females if only nest males participate in the spawning events. However, the fact that egg deposition seems to be mainly induced by males does not support the hypotheses that the prolonged spawning duration is induced by females.

The complete spawning pattern in *L. callipterus *suggests that the male has an overall expectation of the actual egg-laying pattern, and that the exact timing and duration of sperm release are targeted to cumulatively fill the shell with sufficient sperm numbers to fertilize the majority of eggs, taking into account the expected longevity of his sperm. During laying a clutch, males may change strategies of ejaculates as time goes by, because sperm reserves will decrease with increasing number of ejaculates. In *L. callipterus*, males first slightly increase sperm release duration but slightly decrease it during late stage of spawning males, which might also be due to males suffering from sperm shortage (see also [18]). This shows that it is important to know the time since egg laying started, to understand male strategy of ejaculation, and also may explain the variation of ejaculate interval among males.

## Supplementary Material

Supplementary Video: shows spawning behaviour of the cichlid fish *Lamprologus callipterus*, filmed inside a 1000 liter tank at Ethologische Station Hasli (Department of Behavioural Ecology, IEE, University of Bern, Switzerland). The experimental setting of our publication was in smaller 100 liter tanks, with one male, five females and five plexi-glass prepared empty shells (shells of the snail *Neothauma tanganicense*), of which two shells were accessible to the females and could be videotaped.
The first part shows a male at the shell entrance, where a female is currently egglaying deep inside the shell (not visible). The male shows the typical spawning behaviours: (1) male puts his mouth inside the shell (“head-in”); (2) male puts his mouth inside the shell and opens/closes his mouth in rapid sequence, which produces the water current into the shell (“mouthing”); (3) male positions his genital papilla over the shell entrance and releases sperm (“spawning”).
The last part shows a shell prepared with plexi-glass. In the experimental setting of our publication two of these shells where placed close together, always without the blue covering. In this example video, the blue covering was removed when the female had probably almost finished spawning and thereby both the male and the female were disturbed. Nevertheless, the female shows twice “shifting” behaviour, which in our undisturbed experimental setting usually occurred after male mouthing. Clearly visible are the yellow eggs attached to the plexi-glass. Eggs are usually laid singly with a 2 minute interval. The egg can be seen protruding from the female papilla when she moves deep into the shell, after which she carefully attaches the egg to the surface of the plexi-glass or the shell (unfortunately not filmed).

## Figures and Tables

**Table 1:** Spawning behaviours and locations of males and females. (f): Behaviours of which frequencies were recorded, for all other behaviours frequency and duration were recorded.

	Behaviour/location	Description
Male behaviour	Mouthing	The male puts his head into the shell entrance and opens and closes his mouth repeatedly
	Head shake (f)	The male shakes his head quickly side wards in front of the shell entrance
	Head-in (f)	The male puts his head a few mm into the shell entrance but without actively opening and closing the mouth
	Sperm release (i.e. ejaculation)	The male puts his genital papilla over the shell entrance and stays motionless in this position for up to 4 seconds

Female behaviour	Shifting	The female partly moves out of the shell, flickers with the caudal fin and immediately moves back again to her original position. At all times, her tail remains visible and her head remains inside the shell, so no direct visual contact with the male or our fingers appears possible.
	Egg laying (f)	The females deposits an egg at the inner surface of her snail shell
	Moving out of shell	The female comes partly out of the shell

Male location	At the shell	The male is close to the shell (<4 cm)
	Away	The male is >4 cm away from the shell and cannot communicate with the female inside the shell

Female location	In the shell	The female is completely or partly inside the shell^∗^
	Out of the shell	The female is completely out of the shell

*Data analyses were conducted starting when the female was inside the shell.

**Table 2:** Frequencies and proportions of all possible sequences of three essential behaviours of males and females during spawning in the field.

Male mouthing	Female shifting	Male sperm release	Frequency	% of total
Yes	Yes	Yes	473	59.4
Yes	Yes	No	0	0
Yes	No	Yes	3	0.4
Yes	No	No	320	40.2
No	Yes	Yes	0	0
No	Yes	No	0	0
No	No	Yes	0	0

**Table 3:** Changes of the intervals between subsequent egg depositions and sperm releases and sperm release durations in relation to the time course of laying a clutch standardised (time: 0 = first egg laid to 1 = last egg laid). GLM with covariate time (and time squared for egg interval and sperm release duration), random effect of observation number (Nr 1 to 9), and their interaction.

		Egg interval		Ejaculation interval		Sperm release duration	
		(*n* = 946)		(*n* = 2052)		(*n* = 2053)	
	df	*F*	*P*	*F*	*P*	*F*	*P*
Intercept	1	39.5	<0.001	87.4	<0.001	108.4	<0.001
Time	1	14.0	<0.001	1.0	0.32	15.8	<0.001
Time^2^	1	7.5	0.006			9.3	0.002
Nr	8	10.3	<0.001	8.5	<0.001	8.8	<0.001
Nr × time	8	5.5	<0.001	2.0	0.042	4.4	<0.001
Nr × time^2^	8	3.8	<0.001			3.9	<0.001
